# Drug-induced cardiac arrest: a pharmacovigilance study from 2004–2024 based on FAERS database

**DOI:** 10.3389/fcvm.2025.1498700

**Published:** 2025-05-01

**Authors:** Gaocan Ren, Pingping Huang, Jinhui Zhang, Jin Liu, Zian Yan, Xiaochang Ma

**Affiliations:** ^1^ Department of Cardiovascular Disease, Xiyuan Hospital, China Academy of Chinese Medical Sciences, Beijing, China; ^2^ Graduate School, China Academy of Chinese Medical Sciences, Beijing, China; ^3^ Graduate School, Beijing University of Chinese Medicine, Beijing, China; ^4^ Graduate School, Henan University of Chinese Medicine, Zhengzhou, Henan, China; ^5^ National Clinical Research Center for Chinese Medicine Cardiology, Xiyuan Hospital, China Academy of Chinese Medical Sciences, Beijing, China; ^6^ State Key Laboratory of Traditional Chinese Medicine Syndrome, Xiyuan Hospital, China Academy of Chinese Medical Sciences, Beijing, China

**Keywords:** cardiac arrest, FDA, FAERS, adverse events, pharmacovigilance

## Abstract

**Objective:**

Utilizing the FDA Adverse Event Reporting System (FAERS) database, this study conducts signal detection for drugs associated with cardiac arrest (CA), aiming to optimize clinical decision-making and ensure safer drug usage.

**Methods:**

Adverse event reports related to CA from the first quarter of 2004 to the second quarter of 2024 were extracted from the FAERS database. Signal detection was conducted using the reporting odds ratio (ROR) and proportional reporting ratio (PRR) to identify drugs associated with an increased risk of CA.

**Results:**

A total of 66,431 reports were analyzed, comprising 34,508 males (51.9%) and 31,923 females (48.1%). The majority of cases (71.8%) were reported by healthcare professionals, with adults (≥18 years old) representing the predominant group. Clinical outcomes showed that 67.2% of cases resulted in death. Out of 82 drugs with over 100 CA-related reports, 43 displayed positive signals. The top five drugs identified by ROR were: carisoprodol [ROR (95% CI): 34.13 (29.62–39.32)], sugammadex [ROR (95% CI): 26.93 (22.56–32.16)], regadenoson [ROR (95% CI): 20.00 (17.69–22.60)], alprazolam [ROR (95% CI): 12.82 (12.19–13.48)], and propofol [ROR (95% CI): 11.93 (10.61–13.41)]. In the system drug signal detection, musculo-skeletal system drugs ranked highest [ROR (95% CI): 30.99 (27.74–34.62)], followed by alimentary tract and metabolism drugs [ROR (95% CI): 4.75 (4.59–4.92)], nervous system drugs [ROR (95% CI): 4.51 (4.4–4.61)], anti-infective drugs [ROR (95% CI): 4.13 (3.74–4.57)], cardiovascular drugs [ROR (95% CI): 3.89 (3.78–4.01)], and antineoplastic and immunomodulating agents [ROR (95% CI): 2.16 (2.13–2.2)].

**Conclusion:**

This study identifies over 40 drugs potentially associated with an elevated risk of CA based on FAERS data. Healthcare professionals should be particularly vigilant when prescribing these drugs, especially to patients with a history of heart disease, and ensure rigorous monitoring of their cardiac health.

## 1 Introduction

Cardiac arrest (CA), defined as the sudden cessation of mechanical heart function or an effective interruption in blood circulation, is fatal in 90%–95% of cases. Due to its high incidence and mortality rate, it poses a significant challenge in the realm of public health (1–3). Data from Europe and the United States show that the annual incidence of in-hospital cardiac arrest (IHCA) is relatively high, ranging from 1–5 per 1,000 hospitalizations, thereby placing a substantial burden on healthcare systems (4). Although timely cardiopulmonary resuscitation (CPR) improves the likelihood of restoring spontaneous circulation, outcomes remain poor. Specifically, survival rates for out-of-hospital cardiac arrest (OHCA) and IHCA at discharge are only 2%–15% and 15%–22%, respectively (5). Even more concerning is that despite successful CPR, up to 28% of survivors may suffer from severe neurological deficits (6).

CA is a complex event influenced by multiple factors, including ischemic diseases, cardiac arrhythmias, cardiomyopathies, electrolyte imbalances, and age (7). Furthermore, drugs, as a modifiable factor, warrant special attention due to their potential role in precipitating CA (8). Recent research has shown that non-cardiovascular drugs are significantly associated with arrhythmogenic events and CA. For example, a large observational study from Denmark revealed that antiepileptic drugs such as clonazepam and pregabalin significantly increase the risk of CA (9). Eroglu TE et al. examined the link between antidepressant use and CA, finding that high doses of citalopram and escitalopram increased the risk of CA (10). Numerous drugs may heighten CA risk by disrupting the heart's depolarization or repolarization processes (11). However, most of these findings come from observational studies, case reports, and systematic reviews, which often limit the scope of drug investigation and lack comprehensive, real-world exploration of drugs causing CA.

The U.S. Food and Drug Administration's (FDA) Adverse Event Reporting System (FAERS) is a database designed to collect spontaneously reported information on adverse drug events. This database serves for post-market safety surveillance of all FDA-approved drugs, assisting in the detection of potential correlations between drugs and adverse events (AEs), and offering insights into the real-world situation of AEs. Although identifying drugs that cause CA is crucial for ensuring patient safety, there has been a lack of in-depth detection of drug-induced CA risk signals based on the FAERS database. This study aims to analyze drugs related to CA in the FAERS database, to further enhance the safety of drug therapy and reduce the risk of CA events in patients.

## 2 Materials and methods

### 2.1 Data source

The raw data used in this study were collected from 82 quarters (from Q1 2004–Q2 2024) within the FAERS database. Each quarter's dataset contained gender, age, drug administration time, event occurrence time, dosage, causality, and clinical outcomes for individuals reporting adverse reactions. To facilitate efficient analysis, the data from all 82 quarters were merged into a comprehensive summary data sheet (Supplementary Data S1).

### 2.2 Drug standardization and classification

Drug names were extracted from the summary table using the MedEx_UIMA_1.3.8 software developed by Vanderbilt University. A series of processing steps, such as deduplication, coding, and cleaning, were then applied to standardize the drug names (12). Drug classification was performed using the Anatomical Therapeutic Chemical (ATC) classification system (https://www.who.int/tools/atc-ddd-toolkit/atc-classification), established and regularly updated by the World Health Organization Collaborating Centre for Drug Statistics Methodology.

### 2.3 Data filtering

Using the Medical Dictionary for Regulatory Activities (MedDRA) (https://www.meddra.org/), “cardiac arrest” was identified as the preferred term. AEs related to CA were extracted from the FAERS database based on this term. Duplicate reports, defined as entries with identical “PRIMARYID,” reporting dates, drug names, and clinical outcomes, were removed. Drugs associated with CA were categorized into primary suspect (PS), secondary suspect (SS), concomitant, and interaction categories. Only AEs attributed to PS drugs were included in the study. PS drugs with a reporting frequency of over 100 times were selected for further analysis. In addition, when analyzing the occurrence time of adverse reactions, reports without either a “drug start date” or a “drug end date” were excluded, as well as reports with drug start dates later than the reporting time of AEs.

### 2.4 Signal detection method

Disproportionality analysis (DPA), based on a four-fold contingency table (Table 1), was employed to detect AE signals associated with specific drugs. The reporting odds ratio (ROR) and proportional reporting ratio (PRR) (Table 2) were the two primary methods used, both of which are simple to calculate and provide consistent results. Signal identification criteria were as follows: for the ROR method, a positive signal was defined when the number of reported cases (a) was ≥3 and the lower limit of the 95% confidence interval (CI) of the ROR exceeded 1. For the PRR method, a positive signal required that the number of reported cases (a) be ≥3, chi-square (*χ*²) be ≥4, and PRR be ≥2. A signal was only considered valid if both ROR and PRR criteria were met. The strength of the association between a drug and an AE was reflected in the magnitude of the ROR and PRR values, with higher values indicating a stronger correlation. Statistical analysis is completed using R software.

**Table 1:** Four-fold table of disproportionality analysis.

Item	Number of target adverse event reports	Number of other adverse event reports	Total
Target drug	*a*	*b*	*a* + *b*
Other drugs	*c*	*d*	*c* + *d*
Total	*a* + *c*	*b* + *d*	*N* = *a* + *b* + *c* + *d*

**Table 2:** Formulas of reporting odds ratio (ROR) and proportional reporting ratio (PRR).

Methods	Formula	Signal identification criteria
ROR	$\mathrm{ROR}=\frac{\left(a/c\right)}{\left(b/d\right)}=ad/bc$	*a* > 3; A signal is generated if the lower limit of 95% CI of ROR > 1
	$95\text{\%}\;\mathrm{CI}=e^{\mathrm{In}(\mathrm{ROR})\pm 1.96}\sqrt{\frac{1}{a}+\frac{1}{b}+\frac{1}{c}+\frac{1}{d}}$	
PRR	$\mathrm{PRR}=\frac{a(a+b)}{c(c+b)}$	*a* > 3; PRR > 2, χ^2^ > 4
	$\chi^{2}=\frac{{\left(|ab-cd|-N/2\right)}^{2}\times N}{\left(a+b)(a+c)(c+d)(b+d\right)}$	

## 3 Outcomes

### 3.1 Retrieval process

**Figure 1:**
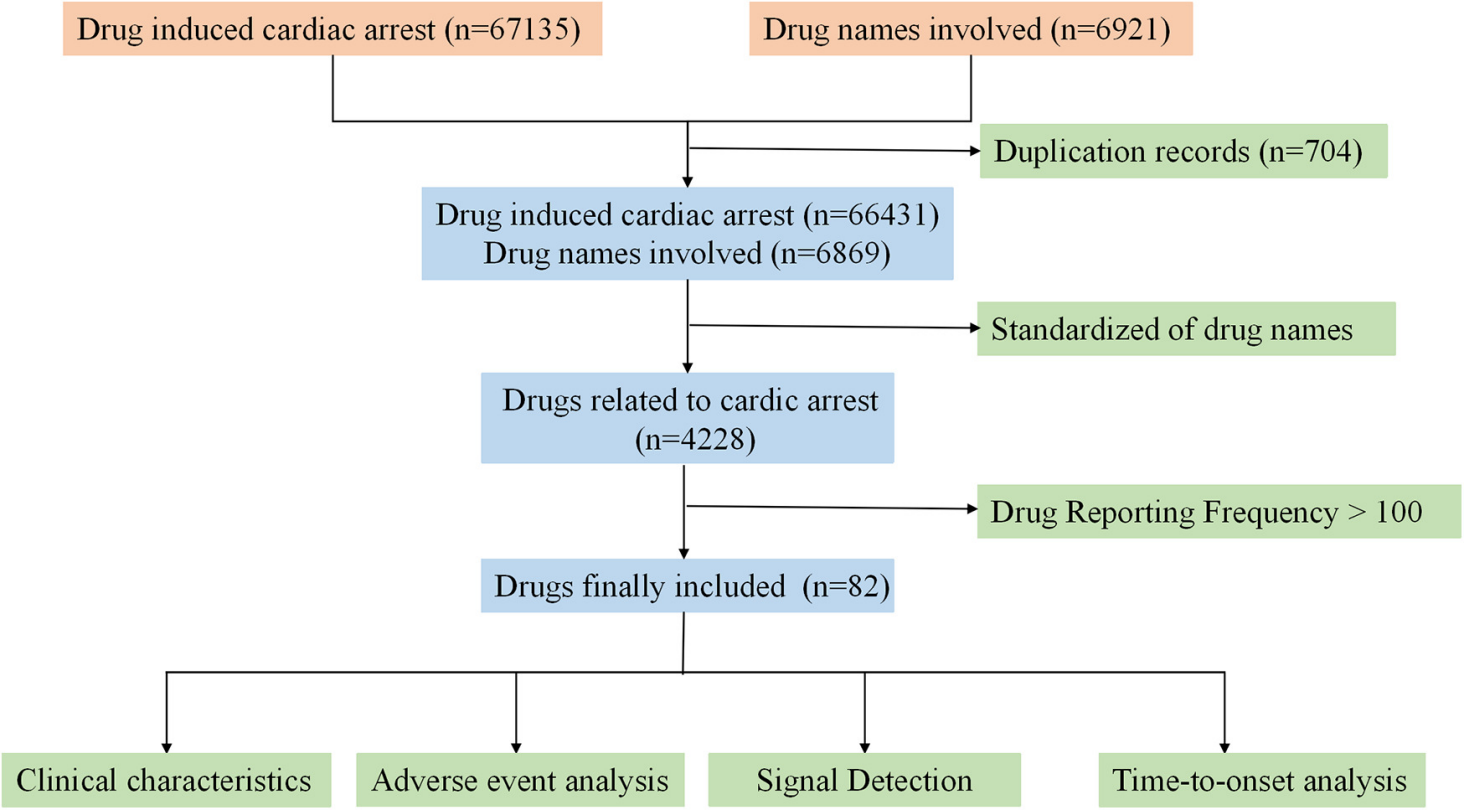
Flowchart for identifying cardiac arrest reports.

A total of 67,135 reports were retrieved from the FAERS database. After removing duplicates and incomplete reports, 66,431 complete reports on CA were collected Following the screening and standardization of drug names, a total of 82 drugs were involved. The specific process is shown in Figure 1.

### 3.2 Basic characteristics of the adverse event reports

#### 3.2.1 Basic information of included reports

This study analyzed 66,431 patient reports. In terms of gender distribution, male patients accounted for a slightly higher proportion, with 34,508 cases (51.9%), compared to 31,923 female patients (48.1%). The majority of cases involved adults (≥18 years old), totaling 53,258 cases (80.1%), highlighting the higher incidence of CA among adults. Reports submitted by healthcare professionals were dominant, with 47,718 cases (71.8%), far exceeding the 13,720 cases (20.7%) reported by consumers, underscoring the credibility and reliability of the data. For more details, refer to Table 3.

**Table 3:** Clinical characteristics of reported cardiac arrest reports.

Dimension	Classification	Number of reports	Percent(%)
Sex			
	Female	31,923	48.1
	Male	34,508	51.9
Age(Year)			
	<18	3,596	5.4
	18–65	33,370	50.2
	≥65	19,888	29.9
	misiing	9,577	14.4
OCCP_COD			
	Physician	23,718	35.7
	Consumer	13,720	20.7
	Other health perfessional	12,562	18.9
	Health perfessional	6,006	9
	Pharmacist	5,397	8.1
	Misiing	4,109	6.2
	Lawyer	884	1.3
	Registered Nurse	35	0.1

#### 3.2.2 Reporting Status of adverse events

**Figure 2:**
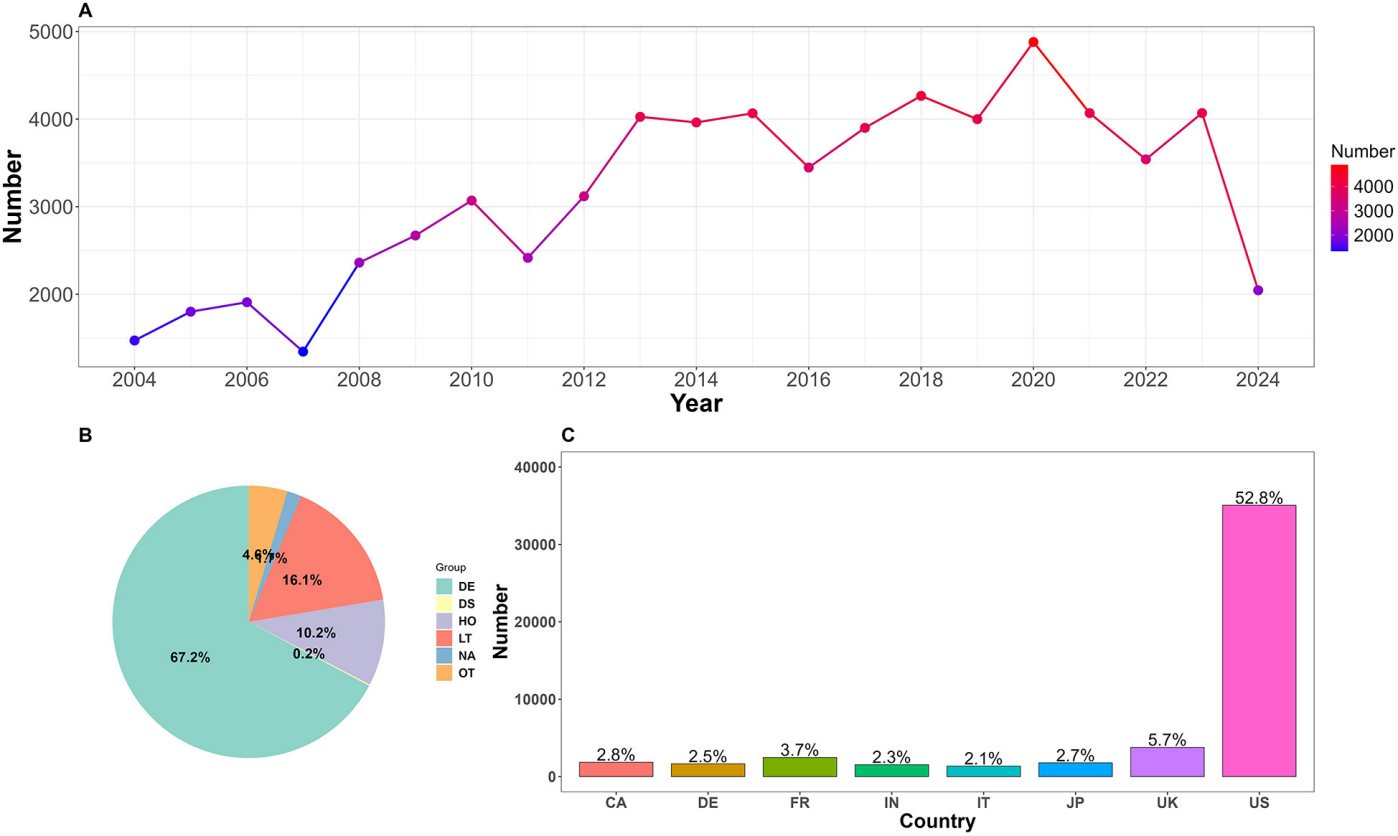
**(A)** Annual number of reported adverse events related to cardiac arrest. **(B)** Proportion of clinical outcomes. DE, death; DS, disability; HO, hospitalization; LT, life-threatening; OT, other; NA, not available. **(C)** Top 8 reporting countries. US, The United States; UK, The United Kingdom; JP, Japan; IT, Italy; FR, France; DE, Germany; CA, Canada.

The number of AEs related to CA exhibited a fluctuating upward trend between 2004 and 2024 (Figure 2A). There was a notable increase after 2007, reaching a peak in 2020 with 4,880 cases. Although there were minor fluctuations subsequently, the overall number remained high, approximately stabilizing at 4,000 cases per year. It is worth noting that the number of AEs in 2024 was lower due to the inclusion of data from only two quarters.

CA-related reports were submitted from 133 countries, with the top eight reporting countries (Figure 2C) being the United States, the United Kingdom, France, Canada, Japan, Germany, India, and Italy, collectively accounting for 74.6% of the total. A total of 2,668 cases (4.01%) did not specify the reporting country.

Due to the FAERS database structure, which allows multiple AEs to be recorded for a single patient, various clinical outcomes were observed. In this study, the most severe outcome was selected as the final outcome. Analysis results (Figure 2B) showed that death was the most prevalent outcome, with 44,665 cases (67.2%). Additionally, 10,701 cases (16.1%) posed a life-threatening situation. Alprazolam reported the highest number of cases among those with a clinical outcome of death. Among cases with life-threatening outcomes, metformin stands out as a new warning signal that deserves attention. Refer to Table 4.

**Table 4:** Top 5 drugs with case outcome of death and life-threatening.

No.	Death			Life-threatening		
	Drug name	Number of reports	Percent(%)	Drug name	Number of reports	Percent(%)
1	Alprazolam	1,367	3	Metformin	152	1.4
2	Rosiglitazone	1,114	2.4	Amlodipine	98	0.9
3	Fentanyl	570	1.2	Ondansetron	97	0.9
4	Lenalidomide	533	1.1	Propofol	93	0.8
5	Adalimumab	446	0.9	Regadenoson	85	0.7

### 3.3 Drugs increasing the risk of cardiac arrest

A total of 4,228 primary suspect (PS) drugs were identified as potentially inducing CA. To ensure robust signal detection, this study focused on 82 drugs with report frequencies exceeding 100. Signal detection using ROR and PRR methods identified 43 drugs with positive signals. These drugs were broadly classified into several categories based on the Anatomical Therapeutic Chemical (ATC) classification system, including alimentary tract and metabolism drugs, blood system drugs, cardiovascular drugs, nervous system drugs, antineoplastic and immunomodulating agents, anti-infective drugs, and musculo-skeletal system drugs. The strength of the risk signals for each drug category was reassessed. All drug signal detection and classifications are provided in Supplementary Table S2.

#### 3.3.1 Single drug signal detection

**Figure 3:**
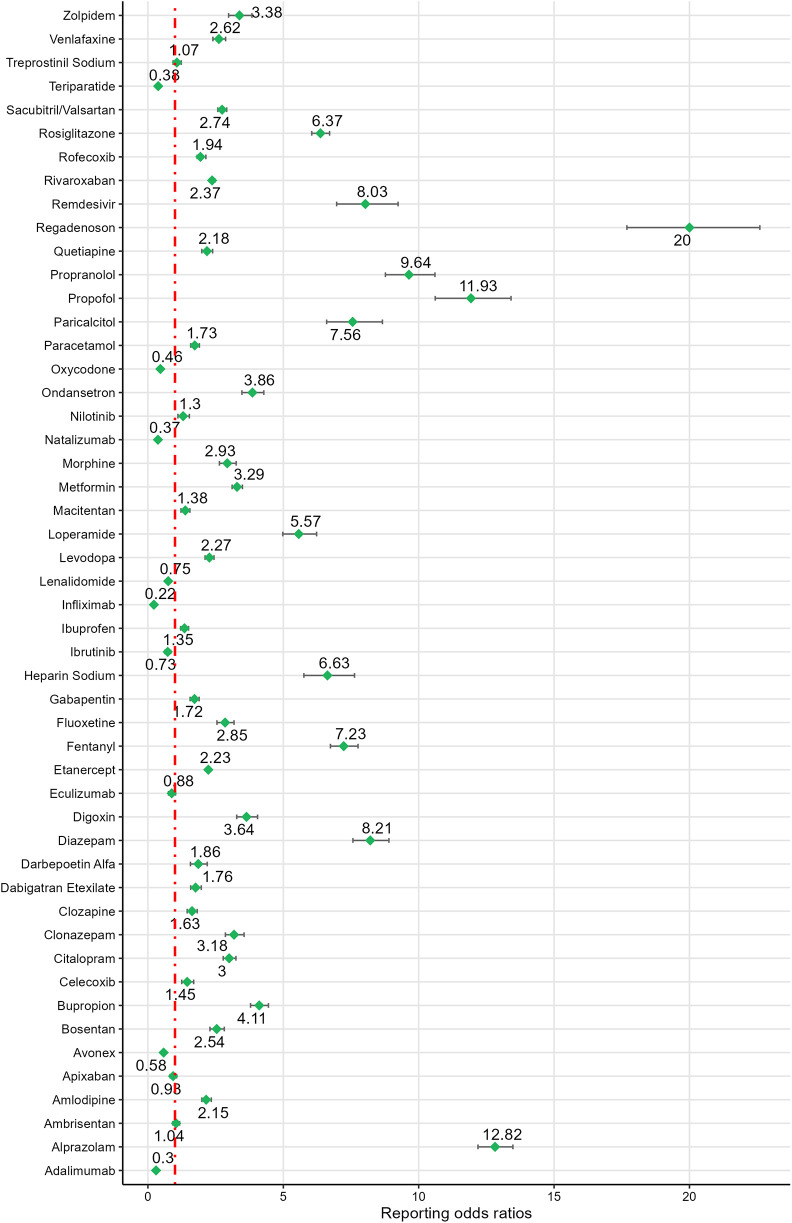
Signal detection results of drugs 1–50.

**Figure 4:**
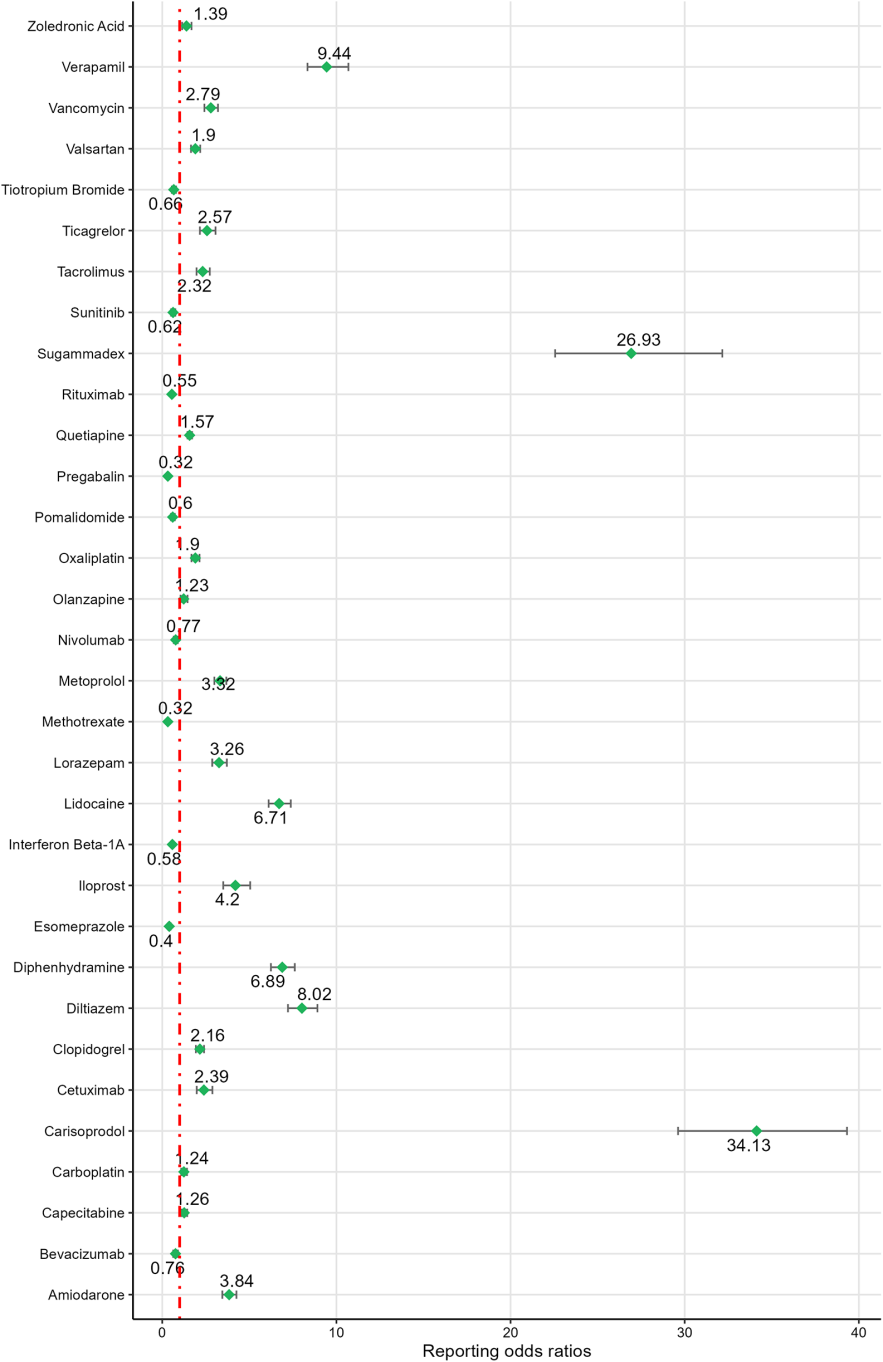
Signal detection results of drugs 51–82.

Based on ROR signal intensity, the top five drugs were: carisoprodol [ROR (95% CI): 34.13 (29.62–39.32)], sugammadex [ROR (95% CI): 26.93 (22.56–32.16)], regadenoson [ROR (95% CI): 20.00 (17.69–22.60)], alprazolam [ROR (95% CI): 12.82 (12.19–13.48)], and propofol [ROR (95% CI): 11.93 (10.61–13.41)]. Other drugs with positive signals, ranked by signal strength, included propranolol, verapamil, diazepam, remdesivir, diltiazem, paricalcitol, fentanyl, diphenhydramine, lidocaine, heparin sodium, rosiglitazone, loperamide, iloprost, bupropion, ondansetron, amiodarone, digoxin, zolpidem, metoprolol, metformin, lorazepam, clonazepam, citalopram, morphine, fluoxetine, vancomycin, sacubitril/valsartan, venlafaxine, ticagrelor, bosentan, cetuximab, rivaroxaban, tacrolimus, levodopa, etanercept, clopidogrel, amlodipine, quetiapine. For detailed information, see Figures 3, 4.

The ROR values for the negative signal drugs were less than 1(Figures 3, 4). The negative signal drugs with ROR values greater than 1 but not meeting the criteria for PRR signal detection include: rofecoxib [PRR(*χ*²): 1.97 (294.97)], dabigatran etexilate [PRR(*χ*²): 1.76 (109.13)], macitentan [PRR(*χ*²): 1.38 (28.87)], clozapine [PRR(*χ*²): 1.63 (71.18)], paracetamol [PRR(*χ*²): 1.73 (134.23)], ambrisentan [PRR(*χ*²): 1.04 (0.32)], gabapentin [PRR(*χ*²): 1.71 (122.15)], treprostinil sodium [PRR(*χ*²): 1.07 (0.84)], ibuprofen [PRR(*χ*²): 1.35 (30.96)], celecoxib [PRR(*χ*²): 1.45 (23.01)], darbepoetin alfa [PRR(*χ*²): 1.85 (55.38)], olanzapine [PRR(*χ*²): 1.23 (6.7)], quetiapine [PRR(*χ*²): 1.57 (119.01)], valsartan [PRR(*χ*²): 1.9 (84.31)], capecitabine [PRR(*χ*²): 1.26 (12.31)], zoledronic acid [PRR(*χ*²): 1.39 (11.27)], carboplatin [PRR(*χ*²): 1.24 (10.86)], nilotinib [PRR(*χ*²): 1.3 (10.12)], and oxaliplatin [PRR(*χ*²): 1.9 (108.89)]. These drugs exhibited no significant signals indicating an increased risk of CA.

#### 3.3.2 System drug signal detection

**Figure 5:**
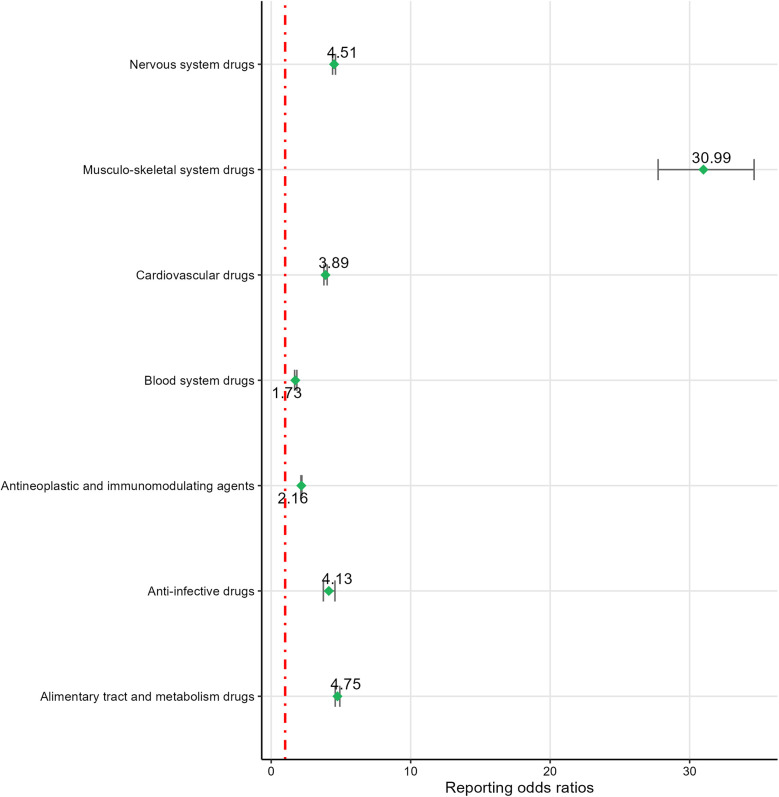
The signal detection of system drugs.

Based on ROR risk signal intensity, musculo-skeletal system drugs ranked highest [ROR (95% CI): 30.99 (27.74–34.62)], followed by alimentary tract and metabolism drugs [ROR (95% CI): 4.75 (4.59–4.92)], nervous system drugs [ROR (95% CI): 4.51 (4.4–4.61)], anti-infective drugs [ROR (95% CI): 4.13 (3.74–4.57)], cardiovascular drugs [ROR (95% CI): 3.89 (3.78–4.01)], and antineoplastic and immunomodulating agents [ROR (95% CI): 2.16 (2.13–2.2)]. Blood system drugs [PRR(*χ*²): 1.73 (305)] were excluded due to not meeting the PRR detection criteria. For detailed information, see Figure 5.

### 3.4 Timing of adverse events

**Figure 6:**
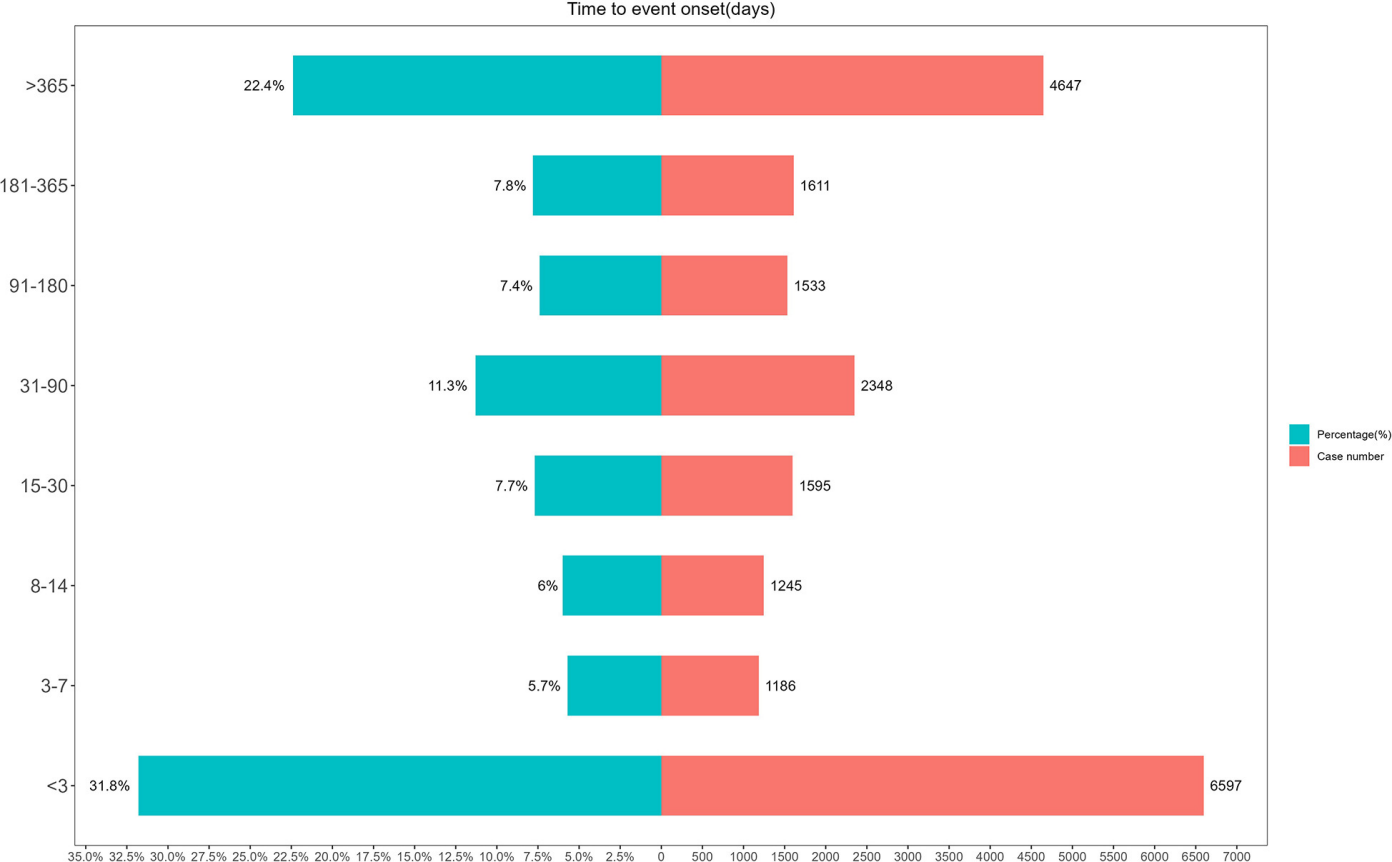
Distribution of time of adverse events.

The timing of AEs (Figure 6) showed that the majority of AEs (37.5%) occurred within seven days, highlighting the importance of prompt detection and early intervention. Furthermore, 22.4% of AEs had a latency period exceeding one year, underscoring the need for long-term monitoring and management of potential risks.

## 4 Discussion

CA, a severe AE, can rapidly lead to death upon onset. However, research specifically focusing on drug-induced CA remains limited. This study utilized data from the FAERS database spanning from Q1 2004 to Q2 2024 to identify high-risk drugs that may trigger CA. The results reveal that drug-induced CA primarily occurs in adult patients, with 67.2% of cases resulting in death and 16.1% posing a life-threatening situation. The majority of reports were submitted by healthcare professionals, with an annual average of approximately 4,000 drug-induced CA cases. These findings emphasize the high mortality rate and frequent occurrence of drug-induced CA, underscoring the urgent need for enhanced drug safety monitoring. Using the ROR and PRR methods, the study successfully identified 43 drugs with positive signals, providing valuable insights for clinical practice and supporting more rational and personalized decision-making.

Musculo-skeletal system drugs showed the highest evaluated risk of CA in this study. Carisoprodol, a meprobamate derivative, acts as a central muscle relaxant (13). Following ingestion, carisoprodol takes effect within 30 min, with muscle relaxation lasting 4–6 h (14). In the United States, a substantial number of AEs have been attributed to the excessive use of carisoprodol, which is considered a significant contributor to accidental deaths (15). While no direct CA cases caused by carisoprodol have been reported, one case involved a male patient who experienced a myocardial infarction (MI) after overdosing on carisoprodol and receiving N-acetylcysteine (16). when carisoprodol is used in conjunction with other drugs, it may serve as a potential risk factor for inducing CA, and therefore requires careful evaluation in clinical practice (17); Sugammadex is a selective muscle relaxant that reverses neuromuscular blockade effectively (18). Boo KY et al. reported a case of a male patient who underwent radiofrequency catheter ablation for atrial fibrillation and, within two minutes of receiving sugammadex postoperatively, exhibited ST-segment elevation on the electrocardiogram, which was followed by CA. The cause was speculated to be associated with coronary artery spasm induced by sugammadex (19). Pereira AV et al. described a 68-year-old male patient who, during an abdominal wall hernia repair surgery, experienced severe bradycardia and hypotension within one minute of sugammadex administration, which was subsequently followed by CA (20). Research indicates that even in patients without pre-existing heart disease, the use of sugammadex may lead to CA, and the severity of this AE may be positively correlated with the drug dose. Therefore, close cardiovascular monitoring is essential following the administration of the drug (21).

This study identified that rosiglitazone and metformin are risk factors for drug-related CA. These two belong to different classes of antidiabetic drugs. Specifically, rosiglitazone belongs to the thiazolidinedione class, and its usage remains controversial. A prospective study indicated that rosiglitazone treatment in patients with type 2 diabetes (T2D) improves cardiovascular (CV) outcomes, reduces the risk of CV death, and is not associated with an increased risk of MI (22). However, multiple studies have indicated that rosiglitazone may elevate CA risk in T2D patients, with CA being one of the composite endpoints for its assessment (23–25). The mechanism behind its correlation with CA might encompass the blockade of cardiac ATP-sensitive potassium (KATP) channels, resulting in delayed afterdepolarizations and triggering fatal arrhythmias (26); There are few recorded cases of metformin-induced CA. Only one case report documented a male patient experiencing CA after consuming 45 g of metformin, potentially attributed to metformin stimulating lactate production in hepatocytes, resulting in metabolic acidosis and subsequently impacting cardiac contractility (27). However, metformin remains a first-line antidiabetic drug. Considering the paucity of reported instances, additional research is necessary to clarify the potential mechanisms underlying metformin-induced CA (28).

Nervous system drugs accounted for 37.2% of drugs with positive signals in this study. Previous studies have noted that nervous system drugs, even at low doses, can trigger severe arrhythmias and increase the risk of CA (29, 30). Anti-anxiety drugs such as diazepam and lorazepam mostly belong to the benzodiazepine class. An observational study of ward patients revealed that for every additional 1 mg of lorazepam consumed, the risk of CA rose by approximately 30% (31). According to the research data, using the defined daily dose (DDD) as the reference benchmark, the use of benzodiazepine drugs presents a dose-dependent risk gradient of CA. Compared with non-users (0 DDD), the hazard ratio (HR) of the group with a daily dose ≤ 1 DDD is 2.64, while the HR of the group with a daily dose > 1 DDD increases to 2.90. In addition, there is also a positive correlation between the duration of drug exposure and the risk of occurrence of CA (32). The mechanism may involve central nervous system depression, hemodynamic instability, and concurrent use with opioids (33); Furthermore, multiple studies have reported cases of CA induced by antidepressants, such as venlafaxine (34) and citalopram (35, 36). Single-time excessive drug intake or long-term administration of high doses of drugs may be the main inducing factors for the onset of the disease (37, 10). Antidepressants can affect cardiac action potentials and prolong the QT interval by inhibiting cardiac ion channels, potentially leading to CA in severe cases (29, 38); As for anesthetic and sedative drugs, they are drugs that necessitate cautious use in clinical settings. Case reports indicate that improper use of propofol can cause hypotension and cardiac conduction abnormalities, thereby increasing the incidence of CA (39, 40). Therefore, in clinical practice, strict control of drug dosages, close monitoring of patient's cardiac function, and avoidance of combinations with other drugs that may increase the risk of CA are essential.

Among anti-infective drugs, remdesivir and vancomycin are associated with a higher risk of CA. Remdesivir, a nucleotide analog prodrug that inhibits viral RNA polymerase, received FDA approval in 2022 for the treatment of COVID-19 (41). In a study investigating the safety of remdesivir in COVID-19 patients, an incidence rate of 3.58% for CA was observed. After controlling for potential confounding factors, it was found that remdesivir significantly increased the risk of CA (OR: 1.88, 95% CI: 1.08–3.29) (42). The metabolite of remdesivir, an adenosine analog, can interact with cardiac A1 receptors, thereby diminishing the autonomy of the atrioventricular node (43). When administered in excessive amounts, it may prolong the QT interval, subsequently triggering severe AEs, including CA (44); Vancomycin belongs to the class of glycopeptide antibiotics. According to a case report, a 9-year-old girl suffered CA after being administered 500 mg of vancomycin via intravenous injection over a span of 5 min. The mechanism underlying this AE may be linked to the induction of Red Man Syndrome (RMS) due to excessively rapid intravenous infusion (45). RMS is essentially an allergic reaction that can trigger skin vasodilation and increased vascular permeability, leading to a reduction in effective circulating blood volume and subsequent hypotension. In severe cases, it may induce CA (46).

Cardiovascular drugs are another major class associated with CA. Amiodarone, a widely recognized antiarrhythmic drug recommended by guidelines, has demonstrated efficacy in the treatment of CA as confirmed by multiple studies (47–49). However, research on the potential risks of amiodarone, such as its ability to inhibit the autonomy of the sinoatrial node, prolong the refractory period, and possibly induce fatal arrhythmias, is currently insufficient (50). Notably, the official product information explicitly lists CA as a potential AE, necessitating strict monitoring of amiodarone usage in clinical practice; Additionally, the use of beta-blockers should be approached with caution. A retrospective study observed a fivefold increase in the risk of CA among patients receiving beta-blocker therapy. This elevated risk is primarily attributed to the potential for conduction delays, bradycardia, and diminished myocardial contractility when beta-blockers are administered in excess, collectively contributing to the occurrence of CA events (51, 52); Lastly, several studies have documented an association between overdoses of calcium channel blockers (particularly non-dihydropyridines) and the occurrence of CA (53, 54). At toxic doses, patients may experience atrioventricular conduction abnormalities, hypotension, and lactic acidosis, conditions that can swiftly escalate and result in CA (55). CA mostly occurs in specific high-risk scenarios, such as the long-term use of amiodarone for non-malignant arrhythmias and the excessive use of β-blockers in decompensated heart failure. In clinical practice, strategies such as strictly grasping the indications, dynamically assessing cardiac function, and strengthening medication monitoring should be adopted to minimize the risks. In emergency situations such as acute myocardial infarction and malignant arrhythmias, the survival benefits of drug interventions significantly outweigh their theoretical risks. For patients with stable cardiovascular diseases, through individualized dose titration and optimized drug selection, the risk of causing CA can be controlled within an acceptable range while ensuring the therapeutic effects.

Tacrolimus, as a potent immunosuppressant, plays a crucial role in organ transplantation and the treatment of autoimmune diseases (56). However, its adverse effects can exacerbate the progression of cardiovascular diseases and trigger severe AEs (57). Reports have suggested that cases of CA within six months after renal transplantation are associated with Tacrolimus (58). Its toxicity frequently leads to renal tubular dysfunction, causing electrolyte disturbances, particularly hyperkalemia, which can result in fatal arrhythmias such as CA in severe cases (59, 60); In the present study, we detected a possible association between Etanercept, a commonly utilized TNF*α* antagonist, and an increased risk of CA. This finding is grounded in our analysis of current data, which indicates an unrecognized potential risk linked to Etanercept use, albeit this has not been substantiated by prior studies; Cetuximab, a targeted therapeutic for cancer, has the potential to elicit severe allergic reactions and infusion-related reactions, which may culminate in CA (61). Furthermore, electrocardiographic abnormalities, including ST-segment depression and inverted T-waves, may manifest during cetuximab administration. However, whether these abnormalities directly precipitate CA necessitates further validation (62). The toxic effects of anti-tumor drugs on cardiac function are mainly manifested as direct damage to cardiomyocytes, cardiac electrophysiological abnormalities, and immune-mediated myocarditis (63). In continuous treatment, a stratified and dynamic strategy should be adopted for cardiac risk assessment: during the baseline period, high-risk populations should be screened through multimodal imaging (echocardiography to assess the ejection fraction and cardiovascular magnetic resonance imaging to detect fibrosis) and biomarkers (troponin, B-type natriuretic peptide). During the treatment, the changes in cardiac function should be monitored every 2–3 cycles, and the drug dosage should be adjusted promptly to reduce the occurrence of CA events.

For patients at high risk of suspected CA, systematic risk assessment and preventive measures should be implemented, including but not limited to the following diagnostic strategies: (1) Screening for arrhythmias (such as QT interval prolongation, Brugada waves, etc.) using a 12-lead electrocardiogram; (2) Cardiac imaging examination (echocardiography to assess cardiac structural abnormalities); (3) Ambulatory electrocardiogram monitoring to capture paroxysmal arrhythmia events. At the same time, great attention should be paid to the risk of drug-induced arrhythmias. A comprehensive review of the drugs currently used by patients that may prolong the QT interval or induce malignant arrhythmias should be carried out, with a focus on verifying the drug dosage, combined use, and treatment course.

To optimize the drug management of patients at high risk of CA, the following strategies are recommended: (1) Individualized risk assessment and dosage adjustment: For patients with structural heart disease, hereditary arrhythmia syndromes, or a history of previous syncope, when taking drugs that may lead to CA, it is recommended to start with the lowest effective dose and establish a strict electrocardiogram monitoring system. If changes in the QT interval and ventricular arrhythmia events occur within 48 h after taking the drug, it is necessary to stop the drug in a timely manner and seek alternative drugs; (2) Prioritize the selection of low-risk drugs: On the premise of meeting the treatment needs, drugs of the same class with a lower risk of arrhythmia should be preferentially selected. For example, cyclobenzaprine can be used as an alternative to carisoprodol, which can effectively avoid the occurrence of CA (64); Neostigmine can replace sugammadex to reverse muscle relaxation and has a smaller impact on the heart (65); (3) Establish an early warning education system: Doctors should formulate a list of drug risk notifications, comprehensively explain to patients the potential CA risks and related symptoms of the drugs they are taking, and enhance patients' awareness of the potential adverse reactions of the drugs. Provide CPR skill training for patients and their families, and emphasize the “golden 4 min” emergency treatment time window.

This study still has several limitations: (1) The underreporting and misreporting of AE reports in the FAERS database may introduce bias into the signal detection results; (2) The population reported in the FAERS database primarily comprises individuals from European and American countries, and ethnic differences could potentially influence the signal detection outcomes; (3) In this study, when analyzing the AE reports, the impact of potential diseases on CA was not taken into account. Additionally, factors such as drug interactions and drug dosages were also not considered. These omissions may lead to biases in the results; (4) The signal detection results can only suggest potential drugs that may increase the risk of AEs, and the causal relationships necessitate further validation through integration with literature and clinical practice. Despite these limitations, this study represents the first comprehensive and systematic signal detection utilizing the FAERS database, providing an exploratory analysis of drugs that may induce CA.

## 5 Conclusion

This study identifies over 40 drugs potentially associated with an elevated risk of CA based on FAERS data. Healthcare professionals should be particularly vigilant when prescribing these drugs, especially to patients with a history of heart disease, and ensure rigorous monitoring of their cardiac health.

## Data Availability

The original contributions presented in the study are included in the article/Supplementary Material, further inquiries can be directed to the corresponding author.
