# Supplementary material for: Drug-induced cardiac arrest: a pharmacovigilance study from 2004–2024 based on FAERS database
Source: Front Cardiovasc Med. 2025 May 1;12:1498700. doi: 10.3389/fcvm.2025.1498700 (PMC12078221; doi:10.3389/fcvm.2025.1498700)
Supplement: Supplementary file 1 [file Table1.docx]

Supplementary Table S4. Single drug signal detection

| Drug name | ROR | lower | Upper |
| --- | --- | --- | --- |
| Rosiglitazone | 6.37 | 6.05 | 6.71 |
| Alprazolam | 12.82 | 12.19 | 13.48 |
| Sacubitril/Valsartan | 2.74 | 2.58 | 2.91 |
| Adalimumab | 0.3 | 0.28 | 0.32 |
| Lenalidomide | 0.75 | 0.7 | 0.81 |
| Rofecoxib | 1.94 | 1.82 | 2.14 |
| Metformin | 3.29 | 3.11 | 3.49 |
| Levodopa | 2.27 | 2.11 | 2.44 |
| Fentanyl | 7.23 | 6.74 | 7.76 |
| Amlodipine | 2.15 | 1.99 | 2.34 |
| Diazepam | 8.21 | 7.57 | 8.9 |
| Bosentan | 2.54 | 2.29 | 2.82 |
| Dabigatran Etexilate | 1.76 | 1.58 | 1.97 |
| Macitentan | 1.38 | 1.22 | 1.55 |
| Clozapine | 1.63 | 1.45 | 1.82 |
| Etanercept | 2.23 | 2.19 | 2.27 |
| Oxycodone | 0.46 | 0.41 | 0.52 |
| Digoxin | 3.64 | 3.28 | 4.05 |
| Ondansetron | 3.86 | 3.47 | 4.28 |
| Avonex | 0.58 | 0.52 | 0.64 |
| Regadenoson | 20 | 17.69 | 22.6 |
| Venlafaxine | 2.62 | 2.4 | 2.87 |
| Apixaban | 0.93 | 0.82 | 1.06 |
| Paracetamol | 1.73 | 1.58 | 1.9 |
| Rivaroxaban | 2.37 | 2.28 | 2.47 |
| Ambrisentan | 1.04 | 0.92 | 1.17 |
| Paricalcitol | 7.56 | 6.6 | 8.66 |
| Gabapentin | 1.72 | 1.56 | 1.89 |
| Fluoxetine | 2.85 | 2.55 | 3.18 |
| Propranolol | 9.64 | 8.77 | 10.6 |
| Natalizumab | 0.37 | 0.32 | 0.42 |
| Citalopram | 3 | 2.78 | 3.25 |
| Treprostinil Sodium | 1.07 | 0.93 | 1.23 |
| Heparin Sodium | 6.63 | 5.76 | 7.63 |
| Clonazepam | 3.18 | 2.86 | 3.55 |
| Ibuprofen | 1.35 | 1.21 | 1.5 |
| Ibrutinib | 0.73 | 0.63 | 0.84 |
| Loperamide | 5.57 | 4.98 | 6.23 |
| Teriparatide | 0.38 | 0.32 | 0.44 |
| Infliximab | 0.22 | 0.19 | 0.28 |
| Celecoxib | 1.45 | 1.25 | 1.69 |
| Propofol | 11.93 | 10.61 | 13.41 |
| Morphine | 2.93 | 2.64 | 3.26 |
| Zolpidem | 3.38 | 2.98 | 3.84 |
| Quetiapine | 2.18 | 1.99 | 2.39 |
| Remdesivir | 8.03 | 6.97 | 9.24 |
| Bupropion | 4.11 | 3.79 | 4.45 |
| Eculizumab | 0.88 | 0.76 | 1.02 |
| Nilotinib | 1.3 | 1.11 | 1.53 |
| Darbepoetin Alfa | 1.86 | 1.57 | 2.19 |
| Oxaliplatin | 1.9 | 1.68 | 2.14 |
| Tacrolimus | 2.32 | 1.97 | 2.73 |
| Amiodarone | 3.84 | 3.45 | 4.26 |
| Clopidogrel | 2.16 | 1.93 | 2.4 |
| Interferon Beta-1A | 0.58 | 0.52 | 0.64 |
| Pregabalin | 0.32 | 0.27 | 0.38 |
| Olanzapine | 1.23 | 1.05 | 1.45 |
| Diphenhydramine | 6.89 | 6.24 | 7.61 |
| Ticagrelor | 2.57 | 2.16 | 3.06 |
| Quetiapine | 1.57 | 1.45 | 1.71 |
| Diltiazem | 8.02 | 7.22 | 8.91 |
| Lorazepam | 3.26 | 2.87 | 3.71 |
| Valsartan | 1.9 | 1.65 | 2.18 |
| Tiotropium Bromide | 0.66 | 0.55 | 0.79 |
| Methotrexate | 0.32 | 0.28 | 0.36 |
| Pomalidomide | 0.6 | 0.5 | 0.72 |
| Bevacizumab | 0.76 | 0.63 | 0.9 |
| Lidocaine | 6.71 | 6.11 | 7.38 |
| Verapamil | 9.44 | 8.34 | 10.69 |
| Nivolumab | 0.77 | 0.66 | 0.89 |
| Sunitinib | 0.62 | 0.51 | 0.75 |
| Capecitabine | 1.26 | 1.11 | 1.43 |
| Vancomycin | 2.79 | 2.42 | 3.2 |
| Zoledronic Acid | 1.39 | 1.14 | 1.68 |
| Cetuximab | 2.39 | 1.98 | 2.88 |
| Iloprost | 4.2 | 3.5 | 5.05 |
| Sugammadex | 26.93 | 22.56 | 32.16 |
| Rituximab | 0.55 | 0.5 | 0.61 |
| Carisoprodol | 34.13 | 29.62 | 39.32 |
| Metoprolol | 3.32 | 2.99 | 3.68 |
| Esomeprazole | 0.4 | 0.34 | 0.48 |
| Carboplatin | 1.24 | 1.09 | 1.42 |

Supplementary Table S4. System Drug Signal Detection

| Drug Classification | ROR | lower | Upper |
| --- | --- | --- | --- |
| Alimentary tract and metabolism drugs | 4.75 | 4.59 | 4.92 |
| Blood system drugs | 1.73 | 1.68 | 1.84 |
| Cardiovascular drugs | 3.89 | 3.78 | 4.01 |
| Nervous system drugs | 4.51 | 4.4 | 4.61 |
| Antineoplastic and immunomodulating agents | 2.16 | 2.13 | 2.2 |
| Anti-infective drugs | 4.13 | 3.74 | 4.57 |
| Musculo-skeletal system drugs | 30.99 | 27.74 | 34.62 |

Supplementary Table S4. Specific Classification of Drugs

| antineoplastic and immunomodulating agents | cardiovascular drugs | Blood system drugs | nervous system drugs | anti-infective drugs | musculo-skeletal system drugs | antineoplastic and immunomodulating agents |
| --- | --- | --- | --- | --- | --- | --- |
| Rosiglitazone | Sacubitril/Valsartan | Rivaroxaban | Venlafaxine | Remdesivir | Sugammadex | Etanercept |
| Metformin | Digoxin | Heparin Sodium | Fluoxetine | Vancomycin | Carisoprodol | Tacrolimus |
| Ondansetron | Regadenoson | Clopidogrel | Citalopram |  |  | Cetuximab |
| Loperamide | Propranolol | Ticagrelor | Bupropion |  |  |  |
| Paricalcitol | Amlodipine | Iloprost | Zolpidem |  |  |  |
|  | Amiodarone |  | Quetiapine |  |  |  |
|  | Diltiazem |  | Alprazolam |  |  |  |
|  | Verapamil |  | Diazepam |  |  |  |
|  | Metoprolol |  | Clonazepam |  |  |  |
|  | Bosentan |  | Lorazepam |  |  |  |
|  | Lidocaine |  | Propofol |  |  |  |
|  |  |  | Levodopa |  |  |  |
|  |  |  | Fentanyl |  |  |  |
|  |  |  | Morphine |  |  |  |
|  |  |  | Diphenhydramine |  |  |  |
